# Young plasma reverses age‐dependent alterations in hepatic function through the restoration of autophagy

**DOI:** 10.1111/acel.12708

**Published:** 2017-12-05

**Authors:** Anding Liu, Enshuang Guo, Jiankun Yang, Yan Yang, Shenpei Liu, Xiaojing Jiang, Qi Hu, Olaf Dirsch, Uta Dahmen, Cuntai Zhang, David A Gewirtz, Haoshu Fang

**Affiliations:** ^1^ Experimental Medicine Center Tongji Hospital Tongji Medical College Huazhong University of Science and Technology Wuhan China; ^2^ Department of Infectious Diseases Wuhan General Hospital Wuhan China; ^3^ Department of Geriatrics Tongji Hospital Tongji Medical College Huazhong University of Science and Technology Wuhan China; ^4^ Experimental Transplantation Surgery Department of General, Visceral and Vascular Surgery Friedrich‐Schiller‐University Jena Jena Germany; ^5^ Department of Pharmacology and Toxicology Massey Cancer Center Virginia Commonwealth University Richmond VA USA; ^6^ Department of Pathophysiology Anhui Medical University Hefei China

**Keywords:** aging, autophagy, hepatocyte, liver injury, liver regeneration, rat, senescence, young blood

## Abstract

Recent studies showing the therapeutic effect of young blood on aging‐associated deterioration of organs point to young blood as the solution for clinical problems related to old age. Given that defective autophagy has been implicated in aging and aging‐associated organ injuries, this study was designed to determine the effect of young blood on aging‐induced alterations in hepatic function and underlying mechanisms, with a focus on autophagy. Aged rats (22 months) were treated with pooled plasma (1 ml, intravenously) collected from young (3 months) or aged rats three times per week for 4 weeks, and 3‐methyladenine or wortmannin was used to inhibit young blood‐induced autophagy. Aging was associated with elevated levels of alanine transaminase and aspartate aminotransferase, lipofuscin accumulation, steatosis, fibrosis, and defective liver regeneration after partial hepatectomy, which were significantly attenuated by young plasma injections. Young plasma could also restore aging‐impaired autophagy activity. Inhibition of the young plasma‐restored autophagic activity abrogated the beneficial effect of young plasma against hepatic injury with aging. *In vitro*, young serum could protect old hepatocytes from senescence, and the antisenescence effect of young serum was abrogated by 3‐methyladenine, wortmannin, or small interfering RNA to autophagy‐related protein 7. Collectively, our data indicate that young plasma could ameliorate age‐dependent alterations in hepatic function partially via the restoration of autophagy.

## 1 INTRODUCTION

As human life expectancy increases, greater numbers of patients are suffering from age‐related diseases. The aging liver is usually associated with alterations of liver morphology and a reduction in liver functions (Schmucker, 2005; Timchenko, 2009). Primary hepatic dysfunctions include a decreased hepatic blood flow, increased accumulation of lipid droplets, impaired liver regeneration, and the development of chronic liver diseases and tumors (Schmucker, 2005; Timchenko, 2009).

Recent studies have indicated that young blood shows therapeutic effect on aging‐associated deterioration of organs. Heterochronic parabiosis is an experimental model, where young and aged mice share a circulation system via the creation of vascular anastomosis by suturing adjacent flanks between the animals. By applying this technique, Conboy et al. found that the muscle and liver from the aged partner of the heterochronic pairs exhibited youthful levels of regeneration (Conboy et al., 2005). A similar “rejuvenating” effect of young blood has been demonstrated in other organs, including the spinal cord (Ruckh et al., 2012), heart (Loffredo et al., 2013), brain (Katsimpardi et al., 2014) (Villeda et al., 2014), β‐cells (Salpeter et al., 2013), and hair follicles (Keyes et al., 2013). Collectively, these observations suggest that exposure to a “young” environment could prevent or reverse age‐dependent decline in the function of critical organ systems.

Autophagy plays an essential role in cellular metabolism and homeostasis by degrading both long‐lived cytoplasmic proteins and dysfunctional organelles via lysosome‐dependent machinery (Klionsky & Emr, 2000; Rautou et al., 2010). Recently, the potential involvement of autophagy in aging and aging‐associated organ injuries has become evident. Defective autophagy reduces the lifespan, whereas induction of autophagy can increase longevity in multiple animal species (Madeo, Tavernarakis, & Kroemer, 2010; Rubinsztein, Marino, & Kroemer, 2011). In aged livers, autophagy activity is decreased, while the restoration of autophagy has been shown to improve cellular maintenance and hepatic function (Schneider et al., 2015; Zhang & Cuervo, 2008).

As young blood can attenuate aging‐associated organ deterioration, and aging is commonly associated with defective autophagy, we hypothesized that young blood could prevent or reverse age‐dependent decline in liver function through the restoration of autophagy. Here we investigated the role of autophagy in the young blood‐afforded beneficial effect against aging‐related alterations in hepatic function.

## 2 RESULTS

### 2.1 Young plasma ameliorates aging‐induced hepatic injury, steatosis, and fibrosis

**Figure 1:**
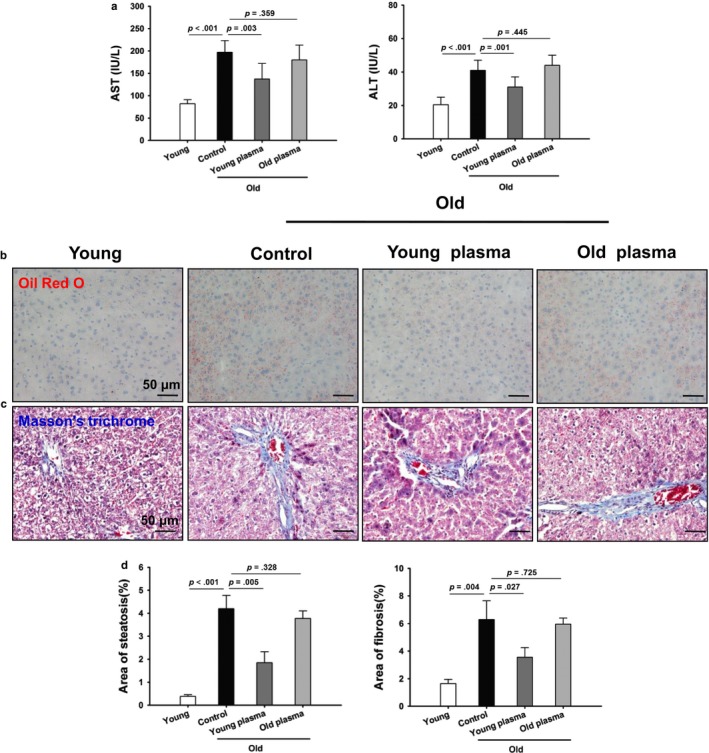
Young plasma improves liver function in aged rats. (a) Serum samples were collected for measuring AST and ALT. The data are shown as mean ± SD, *n* = 8–10 per group. (b) Representative micrographs showing hepatic steatosis in liver sections as stained with Oil Red O (original magnification, 400×). (c) Representative micrographs displaying hepatic fibrosis in liver sections as stained with Masson's trichrome (original magnification, 400×). (D) Quantitative analysis of liver steatosis area and fibrosis area. The data are shown as mean ± SD, *n* = 4 per group

Given that aging induces liver injury and fibrosis, we determined the effect of young plasma administrations on aging‐induced liver injury and fibrosis. As shown in Figure 1a, serum levels of aspartate aminotransferase (AST) and alanine transaminase (ALT) were significantly increased in old rats when compared to those in young controls. However, young plasma administrations significantly decreased serum AST and ALT levels in old rats when compared to those in old plasma‐injected controls. To evaluate the effect of young plasma on aging‐induced hepatic steatosis and fibrosis, Oil Red O and Masson's trichrome staining were performed to examine steatosis and fibrosis, respectively. As shown in Figure 1b–d, the minimal steatosis and fibrosis were noted in young livers. In contrast, both steatosis and fibrosis were increased in aged livers, the effects of which were partially attenuated by young plasma administrations. These data suggest that young plasma may preserve the liver from aging.

### 2.2 Young plasma reduces aging‐induced hepatic senescence and endoplasmic reticulum stress

**Figure 2:**
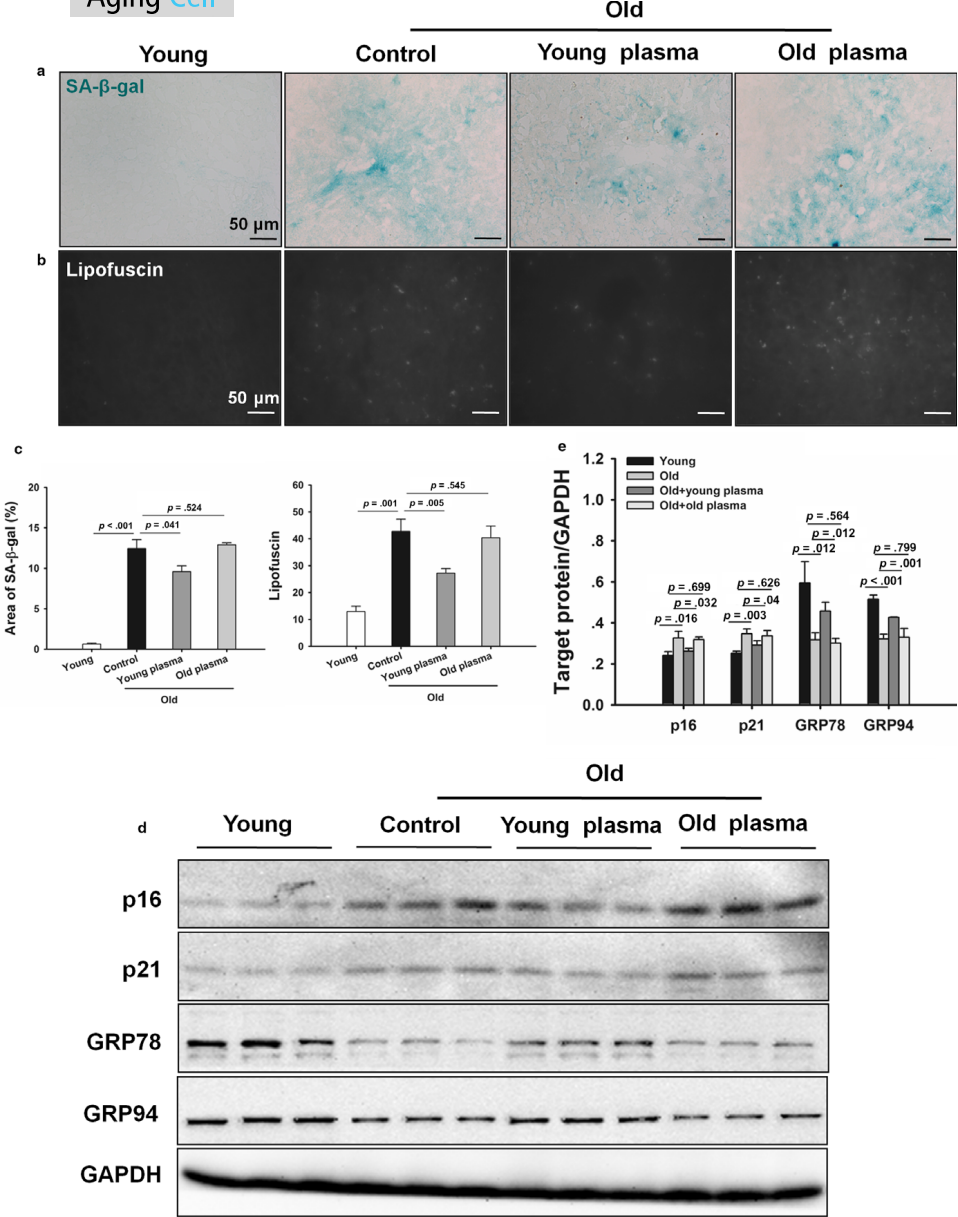
Young plasma prevents hepatic senescence and endoplasmic reticulum stress in aged rats. (a) Representative micrographs showing SA‐β‐gal activity in hepatic tissues (original magnification, 400×). (b) Representative micrographs exhibiting lipofuscin levels in hepatic tissues (original magnification, 400×). (c) Quantitative analysis of the SA‐β‐gal area and lipofuscin levels. The data are shown as mean ± SD. *n* = 4 per group. (d) Representative Western blotting of p16, p21, GRP78, and GRP94 in livers. (e) Densitometric analysis of p16, p21, GRP78, and GRP94. The data are shown as mean ± SD, *n* = 4 per group

As the cellular senescence is characterized by the accumulation of senescence‐associated β‐galactosidase (SA‐β‐gal) activity and lipofuscin, we determined the effects of young plasma on aging‐induced SA‐β‐gal activity and lipofuscin accumulation. SA‐β‐gal activity and lipofuscin accumulation were increased in aged livers as compared to those in young controls. In contrast, young plasma‐treated animals showed less SA‐β‐gal activity and lipofuscin accumulation. This inhibitory effect was not observed in old plasma‐treated rats (Figure 2a–c). In addition, Western blotting analysis showed that the cellular senescence markers including p16 and p21 protein levels were substantially increased in aged livers. The aging‐induced increases in p16 and p21 protein levels were significantly ameliorated by young plasma, but not by old plasma injections (Figure 2d, e). To examine whether young blood could reduce endoplasmic reticulum stress in aged livers, the expression of glucose‐regulated protein 78 (GRP78) and GRP94 was observed. Aging significantly decreased the expression levels of GRP78 and GRP94, while the decline of GRP78 and GRP94 was markedly prevented by young plasma, but not by old plasma injections (Figure 2d, e).

### 2.3 Young plasma restores aging‐induced suppression in liver regeneration

**Figure 3:**
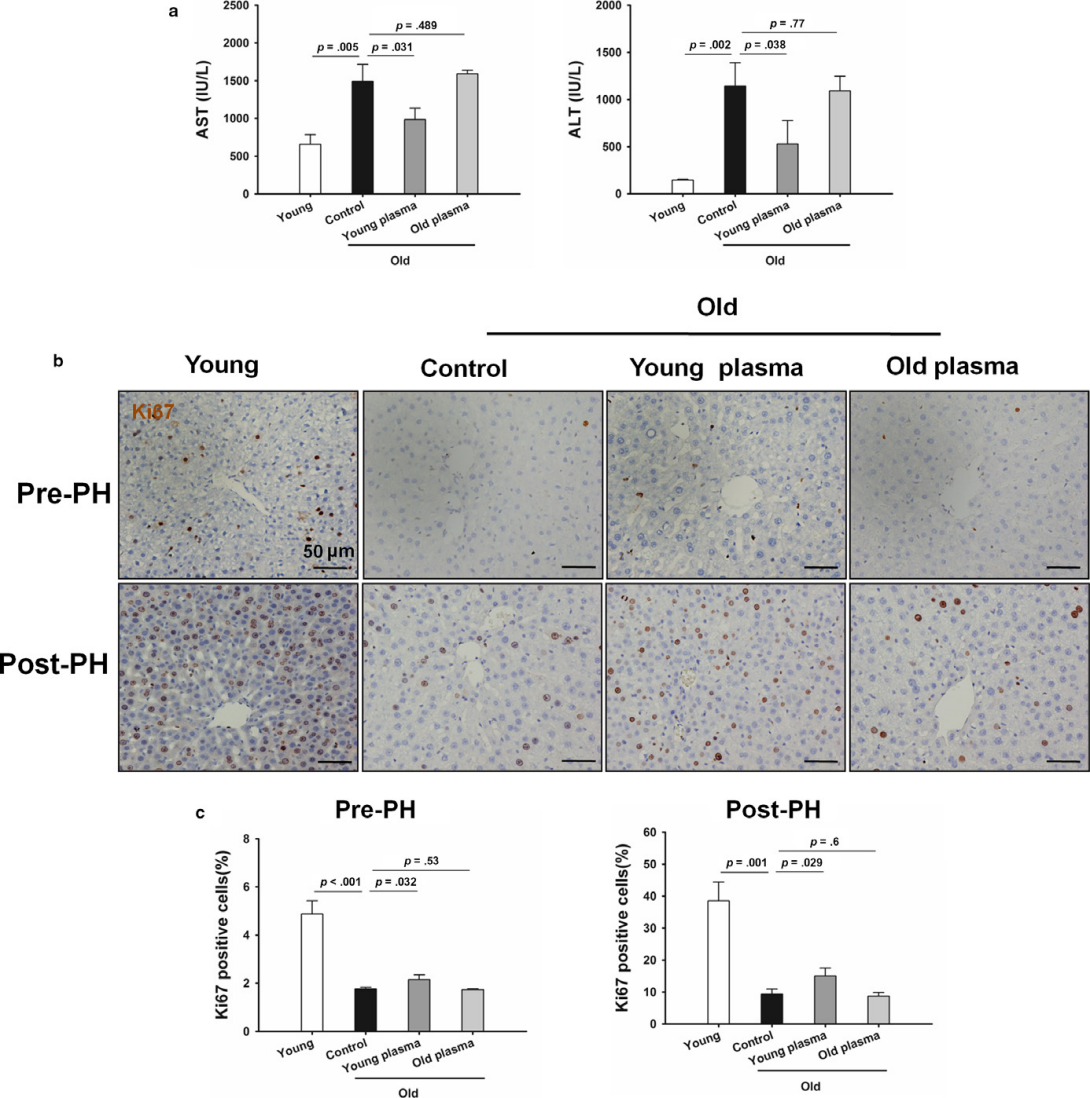
Young plasma rescues defective liver regeneration capacity in aged rats. (a) Quantification of serum AST and ALT levels. The data are shown as mean ± SD, *n* = 4 per group. (b) Representative micrographs exhibiting Ki67‐positive hepatocytes in hepatic tissues obtained from prepartial hepatectomy (PH) and 24 hr post‐PH (original magnification, 400×). (c) The numbers of Ki67‐positive hepatocytes were determined. The data are shown as mean ± SD, *n* = 4 per group

As shown in Figure 3a, the levels of AST and ALT were statistically higher in old rats versus young rats 24 hr after partial hepatectomy. AST and ALT levels were lower in old rats treated with young plasma compared to those in old controls. There were no statistically significant differences in serum levels of AST and ALT between old plasma‐injected rats and old controls. These data showed a decreased hepatocellular damage after partial hepatectomy in old rats treated with young plasma. To determine the role of young plasma in liver regeneration in aged livers, liver regeneration was observed before surgery and 24 hr after operation. Young rats showed higher liver regeneration ability than old rats as indicated by the higher number of Ki67‐positive cells. Furthermore, the number of Ki67‐positive cells was statistically higher in old rats treated with young plasma both before surgery and 24 hr after operation than in old controls (Figure 3b, c).

### 2.4 Young plasma ameliorates liver aging via restoration of autophagy

**Figure 4:**
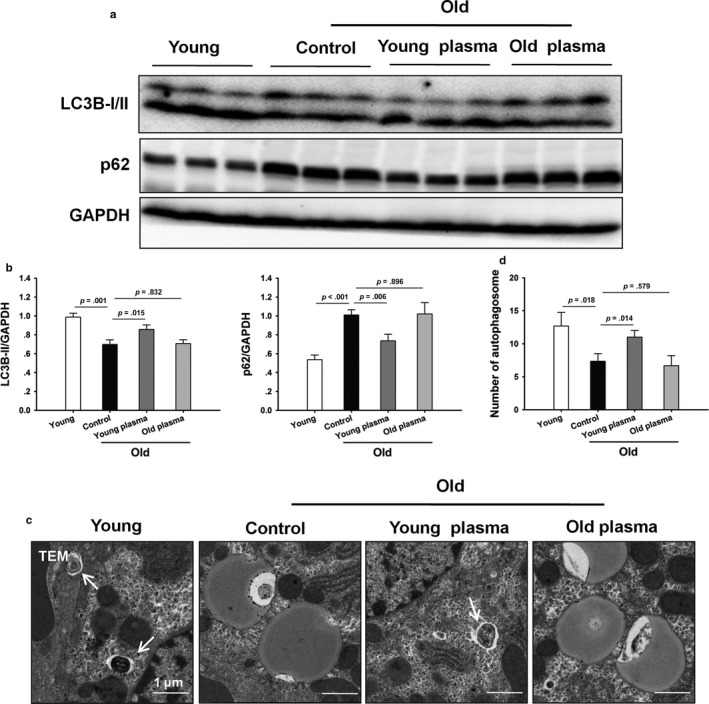
Young plasma restores aging‐induced suppression in hepatic autophagy. (a) Representative Western blotting of LC3B and p62. (b) Densitometric analysis of LC3B‐II and p62. (c) Representative transmission electron microphotographs showing autophagosomes in hepatic tissues (original magnification, 5000×). Autophagosomes are indicated by arrows. (d) The numbers of autophagosomes were calculated. The data are shown as mean ± SD, *n* = 4 per group

To study the effect of autophagy on young plasma‐afforded protection against hepatic injury with aging, the expression pattern of microtubule‐associated protein 1 light chain 3B (LC3B) was first determined. Aging markedly decreased hepatic protein expression of LC3B‐II. LC3B‐II protein expression was decreased in the liver tissue of aged rats compared to the young controls and young plasma restored the aging‐induced loss in LC3B‐II. Aging also markedly elevated p62 protein expression in the liver, while young plasma markedly attenuated p62 protein expression in the old rats (Figure 4a, b). This observation was confirmed by the transmission electron microscopy (TEM) analysis of autophagosome. In young rats, the number of autophagosomes was statistically higher in livers than those in the old rats. Moreover, young plasma partially restored the aging‐induced loss of autophagosomes (Figure 4c, d). Furthermore, young plasma restored the aging‐impaired autophagic flux, as shown by marked increase in LC3B‐II and p62 expression in aged livers by chloroquine (Fig. S1).

**Figure 5:**
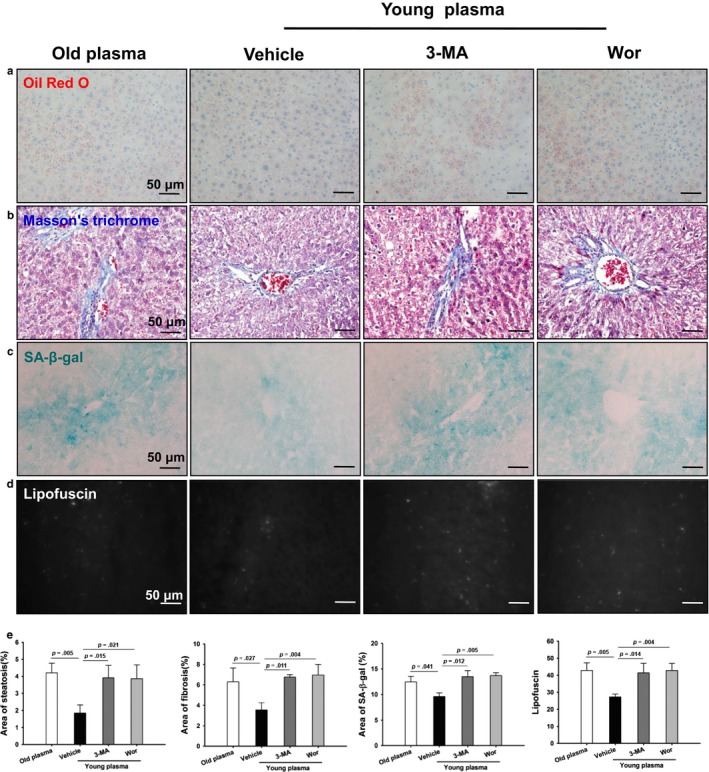
Young plasma prevents liver aging via the restoration of autophagy. Aged rats were treated with pooled young plasma (1 ml, IV) three times per week for 4 weeks. 3‐methyladenine (3‐MA, 30 mg/kg, IP) or wortmannin (Wor, 0.6 mg/kg, IV) was then given to the rats 3 times per week for 2 weeks before harvest. (a) Representative micrographs showing hepatic steatosis in liver sections as stained by Oil Red O (original magnification, 400×). (b) Representative micrographs displaying hepatic fibrosis in liver sections as stained with Masson's trichrome (original magnification, 400×). (c) Representative micrographs showing SA‐β‐gal activity in hepatic tissues (original magnification, 400×). (d) Representative micrographs exhibiting lipofuscin levels in liver sections (original magnification, 400×). (e) Quantitative analysis of the liver steatosis area, fibrosis area, SA‐β‐gal area, and lipofuscin levels. Data are shown as mean ± SD, *n* = 3 per group

To further evaluate the contribution of autophagy to the young plasma‐induced beneficial effect of hepatic injury with aging, rats were pretreated with the pharmacological autophagy inhibitor 3‐methyladenine (3‐MA) or wortmannin, respectively. The 3‐MA or wortmannin treatment led to a reduction in LC3B‐II protein expression (Fig. S2). Cellular senescence markers including p16 and p21 protein levels were substantially increased in the 3‐MA‐ or wortmannin‐treated rats (Fig. S2). Of note, steatosis, fibrosis, SA‐β‐gal activity, and lipofuscin accumulation in the 3‐MA‐ or wortmannin‐treated rats were significantly higher than those in the vehicle controls (Figure 5a–e). The livers of 3‐MA‐ or wortmannin‐treated rats also exhibited elevated endoplasmic reticulum stress as evident by inhibition of GRP78 and GRP94 protein expression (Fig. S2). Furthermore, the number of Ki67‐positive cells was statistically lower in the livers from 3‐MA‐ or wortmannin‐treated rats (Fig. S3).

### 2.5 Young serum reduces cell senescence via restoration of autophagy in primary hepatocytes

**Figure 6:**
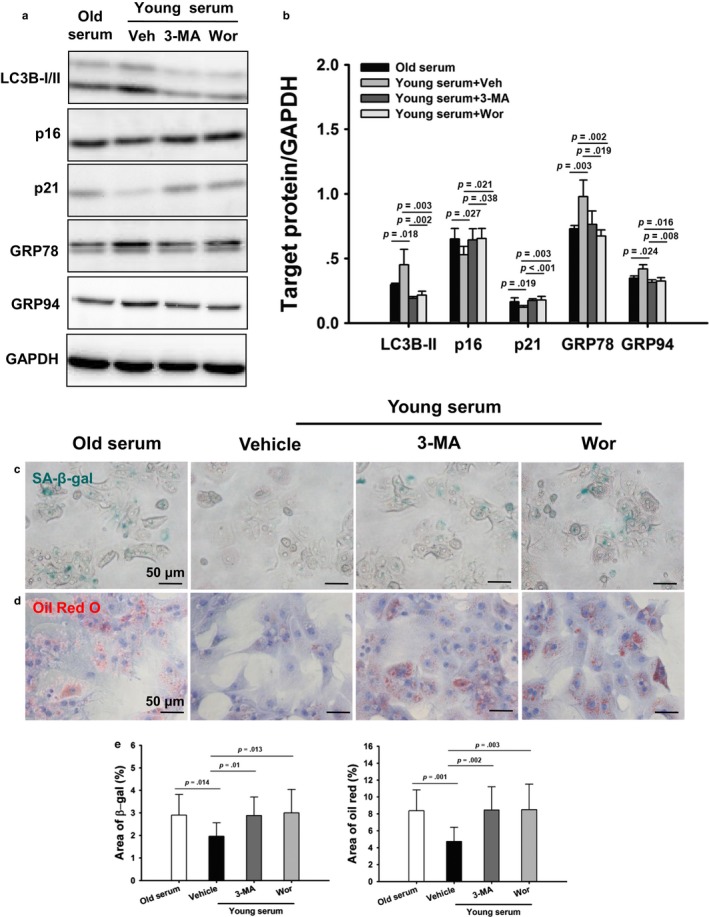
Inhibition of autophagy abrogates the antisenescence effect of young serum. Old hepatocytes were treated with young serum in the absence or presence of vehicle (Veh), 3‐MA (10 mM), or wortmannin (Wor, 1 μM) for 72 hr. (a) Representative Western blotting of LC3B, p16, p21, GRP78, and GRP94 protein expression. (b) Densitometric analysis of LC3B‐II, p16, p21, GRP78, and GRP94. The experiment was performed in triplicate with similar results. The data are shown as mean ± SD. (c) Representative micrographs of SA‐β‐gal staining in hepatocytes with or without young serum, 3‐MA, or Wor (original magnification, 400×). (d) Representative micrographs of Oil Red O staining in hepatocytes in the absence or presence of young serum, 3‐MA, or Wor (original magnification, 400×). (e) Quantitative analysis of SA‐β‐gal and lipid accumulation. The experiment was performed in triplicate with similar results. The data are shown as mean ± SD

To further address the role of autophagy in young plasma‐induced beneficial effect on hepatic senescence, autophagy was blocked by 3‐MA, wortmannin, or small interfering RNA (siRNA) to autophagy‐related protein 7 (Atg7) in cultured old hepatocytes, respectively. Young serum significantly increased autophagic flux as shown by higher expression levels of LC3B‐II and p62 expression in old hepatocytes by chloroquine (Fig. S4). Young serum significantly increased LC3B‐II, GRP78, and GRP94 protein levels, and decreased p16 and p21 protein levels, which were prevented by 3‐MA or wortmannin treatment (Figure 6a, b). Furthermore, aging dramatically increased the accumulation of SA‐β‐gal in hepatocytes, which was markedly decreased by young serum. Young serum also reduced lipid accumulation as seen by Oil Red O staining. In contrast, inhibition of autophagy with 3‐MA or wortmannin abrogated the inhibitory effects of young serum on β‐gal and lipid accumulation (Figure 6c–e). Consistent with 3‐MA or wortmannin, autophagy inhibition by Atg7 knockdown abrogated the antisenescence effect of young serum (Fig. S5).

## 3 DISCUSSION

Aging lowers the function of the liver in both rodents and humans. Exposure to “young” circulation could rejuvenate aged cells activity in several organs. The mechanisms responsible for this phenomenon are still poorly understood. Here we demonstrated that young plasma could attenuate the age‐dependent alterations in hepatic function. Importantly, autophagy activity was impaired in aged livers, and the restoration of autophagy activity by young plasma may act as a key mechanism for its beneficial effect against liver aging.

Aging liver is associated with alterations of morphology and biology that lead to hepatic injury. Lipofuscin, an autofluorescent compound, is accumulated in aging liver (Zhang & Cuervo, 2008). More importantly, lipofuscin has been shown to influence cellular function including cellular adaptability and lysosome function (Neufeld, 1991; Terman, Dalen, & Brunk, 1999). Furthermore, lipofuscin decreases survival time and inhibits autophagy in human fibroblasts during amino acid starvation (Terman et al., 1999). Conversely, inhibition of autophagy leads to an impaired turnover of lysosomes and accumulation of lipofuscin‐like material in human fibroblasts (Stroikin, Dalen, Loof, & Terman, 2004). In our study, young plasma significantly prevented cellular lipofuscin and SA‐β‐gal accumulation in aged livers, supporting a protective role of young blood against aging in the liver.

Aging also increases the accumulation of lipid droplets in the cytoplasm of hepatocytes (Kuk, Saunders, Davidson, & Ross, 2009). Evidence is also available indicating that excessive steatosis and fibrosis dramatically inhibited hepatic function in various pathological conditions including aging (Kuk et al., 2009). More importantly, autophagy has been reported to be linked to lipid metabolism (Liu & Czaja, 2013; Liu, Guo, et al., 2016; Singh et al., 2009; Yang, Li, Fu, Calay, & Hotamisligil, 2010). Intracellular lipids regulate levels of autophagy, and inhibition of autophagy increases hepatic lipid storage in cultured hepatocytes and mouse liver during starvation (Liu & Czaja, 2013). In our study, the overt steatosis and fibrosis in aged livers were markedly attenuated by young plasma injections, suggesting that young blood may preserve the liver from fibrosis in aging.

Aging reduces the ability of the liver to regenerate. Liver regeneration is impaired in old animals and in elderly humans (Schmucker & Sanchez, 2011; Takubo et al., 2000; Timchenko, 2009). Furthermore, a recent study demonstrated that autophagy plays a critical role in liver regeneration and may be essential for the preservation of cellular quality by preventing hepatocytes from becoming senescent and hypertrophic (Toshima et al., 2014). Importantly, Conboy et al. reported that a significant increase in the proliferation of the aged hepatocyte progenitors in the liver from the aged partner of the heterochronic pairs (Conboy et al., 2005). In agreement with these studies, we demonstrated that the regenerative capacity was impaired in aged livers, and young plasma restored liver regeneration capacity in aged livers after partial hepatectomy, indicating that young plasma may reverse the age‐associated decline in the capacity of the liver to regenerate.

Perhaps the most intriguing finding of our study was that young plasma could restore aging‐impaired autophagy. Reduced autophagy has been associated with accelerated aging, whereas induction of autophagy might delay aging and extend longevity (Madeo et al., 2010; Rubinsztein et al., 2011). In addition, restoration of autophagy in aging liver has been shown to decrease the age‐associated stigmata, including a decrease in oxidized proteins abundances and the reduction in polyubiquitinated protein aggregates and apoptotic cells (Zhang & Cuervo, 2008). Based on these findings, we hypothesized that young blood could reverse age‐dependent alterations in hepatic function via the restoration of autophagy. Our data showed that autophagy was impaired in aged livers. More importantly, administration of young plasma could restore autophagy activity. To determine the involvement of an autophagic mechanism in the protection offered by young plasma, young plasma‐induced autophagy was inhibited using 3‐MA or wortmannin. As expected, both 3‐MA and wortmannin administration abolished the anti‐aging effect of young plasma. This finding was further consolidated by the data from *in vitro* studies, which showed that young serum prevented old hepatocytes from senescence and treatment with 3‐MA, wortmannin, or Atg7 siRNA abrogated the antisenescence effect of young serum, respectively. These investigations suggest the protective role of young plasma on aging‐induced hepatic injury may be partially via the restoration of autophagy.

How does autophagy ameliorate liver aging? Recent studies have demonstrated that the anti‐aging effect of autophagy is related to its cell‐autonomous functions, which avoids accumulation of toxic proteins (e.g., misfolded or aggregated proteins) and organelles (e.g., damaged mitochondria) and inhibits cell death (Mizushima & Komatsu, 2011; Rubinsztein et al., 2011). Furthermore, autophagy may also mediate anti‐aging effect by cell‐nonautonomous effects, including the clearance of intracellular pathogens, the reduction in inflammatory cytokine secretion, and the improvement of neuroendocrine circuits function (Mizushima & Komatsu, 2011; Rubinsztein et al., 2011). We demonstrated that young plasma could restore autophagy activity in aged livers. Autophagy may ameliorate liver aging via both its cell‐autonomous and cell‐nonautonomous functions *in vivo*, and reduce cell senescence through its cell‐autonomous functions in primary hepatocytes.

Cellular senescence is a terminal arrest of proliferation, which triggered by various cellular stresses including telomere dysfunction, DNA damage, and genotoxic and oxidative stress (Gewirtz, 2013; Jeyapalan & Sedivy, 2008; Kang & Elledge, 2016). Senescent cells accumulate in various tissues and organs with aging (Campisi, 2005). Senescent cells have been demonstrated to disrupt tissue structure and function through the secretion of pro‐inflammatory cytokines, chemokines, and proteases, a feature termed the senescence‐associated secretory phenotype (Campisi & d'Adda, 2007; Vicente, Mausset‐Bonnefont, Jorgensen, Louis‐Plence, & Brondello, 2016). Recent studies have demonstrated that selectively eliminating senescent cells can attenuate several age‐dependent disorders (Baker et al., 2011; Chang et al., 2016; Roos et al., 2016; Zhu et al., 2015). The relationship between autophagy and senescence is still subject to debate (Kang & Elledge, 2016). Autophagy acts in one instance to promote senescence but in another to prevent it (Gewirtz, 2013; Kang & Elledge, 2016). Our data suggest that restoration of autophagy could inhibit cellular senescence.

In summary, aging impaired hepatic autophagy activity and subsequently led to liver injury. Young plasma could partially restore the defective autophagy activity and alleviate aging‐induced hepatic injury, favoring a prominent role of autophagy in young plasma‐induced beneficial effect against liver aging. These findings suggest that young blood may afford a therapeutic potential role in the management of aging‐associated deterioration of organs.

## 4 EXPERIMENTAL PROCEDURES

### 4.1 Experimental design

The experiments were designed to investigate whether young blood could reverse aging in the liver via the restoration of autophagy both in aged rats and primary old hepatocytes. To determine whether young blood could reverse aging in the liver, aged rats were treated with pooled plasma (1 ml, intravenously [IV]) collected from young (3 months, *n* = 200) or aged (22 months, *n* = 80) rats three times per week (Monday, Wednesday, and Friday) for 4 weeks. Serum AST and ALT levels, SA‐β‐gal activity, lipofuscin deposits, lipid accumulation, fibrosis, and liver regeneration were analyzed. To investigate whether young blood could protect against aging in the liver via the restoration of autophagy, 3‐MA (30 mg/kg, intraperitoneally [IP], Cayman Chemical, 13242) or wortmannin (0.6 mg/kg, IV, Cayman Chemical, 10010591) was given to rats three times per week (Tuesday, Thursday, and Saturday) for 2 weeks before sacrifice to inhibit young plasma‐induced autophagy, respectively. To determine whether young blood could inhibit primary old hepatocyte senescence, SA‐β‐gal activity and lipid accumulation were investigated 72 hr after treatment with either young serum or old serum. To investigate whether the antisenescence effect of young serum was autophagy dependent, 3‐MA (10 mM), wortmannin (1 μM), or Atg7 siRNA (50 nM, Life Technologies, 259550) was used to inhibit young blood‐induced autophagy.

### 4.2 Experimental animals

Young male inbred Sprague Dawley rats (3 months, weighing 200–250 g) and aged rats (22 months, weighting 600–800 g) were housed under standard animal care conditions and had free access to food and water. This study was approved by the Institutional Animal Care and Use Committee of Huazhong University of Science and Technology and followed the National Institutes of Health guidelines for animal care.

### 4.3 Plasma and serum collection

Pooled plasma was collected from young (3 months) or aged (22 months) rats, respectively. Rats were completely anesthetized with pentobarbital (60 mg/kg, IP). After opening the abdomen, a blood sample was collected from the inferior vena cava. Plasma and serum were obtained from blood with or without heparin by centrifugation at 1,000 *g* and 5 min. All plasma and serum aliquots were stored at −80°C until use. Old rats were treated with pooled plasma (1 ml, IV) collected from young or aged rats three times per week (Monday, Wednesday, and Friday) for 4 weeks.

### 4.4 Partial hepatectomy model

A 70% partial hepatectomy model was performed as described previously (Madrahimov, Dirsch, Broelsch, & Dahmen, 2006). Briefly, rats (*n* = 4) were completely anesthetized with pentobarbital. After opening the abdomen and dissecting interlobular ligaments, the left lateral lobe and median lobe were resected. Rats were sacrificed 24 hr after the operation to evaluate liver regeneration.

### 4.5 Primary hepatocyte isolation and culture

Primary rat hepatocytes were isolated and cultured by a modified *in situ* collagenase perfusion technique as described (Kim, Qian, & Lemasters, 2003; Liu, Guo, et al., 2016; Liu, Huang, et al., 2016). Briefly, after anesthetizing with pentobarbital, liver was perfused *in situ* with warm Hank's Balanced Salt Solution (Life Technologies, 14175) via the portal vein and was followed by liver warm basic Dulbecco's Modified Eagle's Medium (DMEM, Life Technologies, 11995) containing 0.75 mg/ml collagenase D (Sigma‐Aldrich, C5138) for 10–15 min. The liver was excised and minced in basic DMEM medium. The cell suspension was filtered through a 70‐μm nylon mesh (Corning Life Sciences, 352350). The filtrate was centrifuged at 30 g for 5 min at 4°C, and the pellet was washed 3 times with basic DMEM medium. The viability of hepatocytes was 95% as judged by trypan blue exclusion. The hepatocytes were cultured on plates coated with rat tail collagen (Sigma‐Aldrich, C3867) in Opti‐MEM I reduced serum medium (Life Technologies, 31985) supplemented with 5% serum from young or old rats.

### 4.6 Liver damage assessment

Blood was drawn from the inferior vena cava and centrifuged at 3,000 *g* for 5 min. The serum levels of AST and ALT were determined using an automated chemical analyzer (Hitachi Co., Tokyo, Japan).

### 4.7 Lipofuscin autofluorescence

Liver samples were embedded in optimal cutting temperature medium (OCT, Sakura Finetek, 4583) and frozen in liquid nitrogen. Cryosections were cut at 7 μm thickness using a Leica cryomicrotome (Leica Microsystems, Buffalo Grove, IL). After washing with phosphate‐buffered saline (PBS, Life Technologies, 10010), sections were air‐dried and then mounted in fluorescent mounting medium. Autofluorescent lipofuscin deposits were visualized using an Olympus BX‐51 microscope (Olympus, Tokyo, Japan) through the FITC light channel and were counted in 5 fields/section (×400). The amount of lipofuscin/field was shown.

### 4.8 Oil Red O staining

Cryosections (7 μm thick) or cells were fixed in 10% formalin for 10 min. The slides or cells were incubated with freshly prepared Oil Red O working solution for 15 min and then counterstained with hematoxylin for 5 min. Lipid accumulation was digitalized using an Olympus BX‐51 light microscope, and the Oil Red O‐stained area was determined measured with the ImageJ software (NIH, Bethesda, MD).

### 4.9 Masson's trichrome staining

Formalin‐fixed liver tissues were embedded in paraffin and sliced into 4‐μm‐thick sections. Sections were stained with Masson's trichrome staining. Fibrosis was determined by calculating the percentage of the total hepatic area using the ImageJ software.

### 4.10 Immunohistochemistry

Immunohistochemical staining was performed in a Leica Bond Max automated system (Leica Biosystems, Nussloch, Germany) using the Leica‐Refine detection kit (Leica Biosystems, DS9800). Briefly, after de‐paraffinization, rehydration, and antigen retrieval, the sections were then incubated with Ki‐67 antibody (1:200, Abcam, ab16667) for 30 min at room temperature, followed by detection using the DAB system. Ki67‐positive cells were counted in 5 fields/section under a microscope (400 x), and the number of cells/field was shown.

### 4.11 TEM analysis

For electron microscopy, liver tissues (1 mm^3^ in size) were fixed with 2% paraformaldehyde and 2.5% glutaraldehyde in 0.1 m sodium cacodylate buffer and processed for TEM as described previously (Liu, Fang, Wei, Dirsch, & Dahmen, 2014). Ultrathin sections (80 nm) were cut and stained with uranyl acetate followed by lead citrate and observed on a Tecnai G2 electron microscope (FEI Electron Optics, Eindhoven, The Netherlands). For quantification, the total amount of autophagic vacuoles was calculated from 10 micrographs taken randomly.

### 4.12 siRNA Transfection

Transfection of siRNA against rat Atg7 was conducted using Lipofectamine RNAiMAX reagent (Invitrogen, 13778–075) following the manufacturer's guidance. A scrambled siRNA was transfected as negative control. After transfection for 48 hr, hepatocytes were treated with different conditions for further analysis.

### 4.13 SA‐β‐gal staining

This assay was performed using a commercially available senescence detection kit (BioVision, K320–250). Briefly, cryosections and cells were fixed with fixative solution for 10–15 min at room temperature, followed by staining with fresh β‐gal staining solution overnight at 37°C. The β‐gal‐stained area was measured with ImageJ software.

### 4.14 Western blotting analysis

Liver lysates and serum samples were prepared, and Western blotting was performed as previously described (Liu et al., 2014). Proteins were resolved with sodium dodecyl sulfate–polyacrylamide gel electrophoresis followed by transferred to polyvinylidene difluoride membranes. The membranes were incubated overnight with primary antibody at 4°C. Polyclonal rabbit anti‐LC3B (1:1000, Abcam, ab48394), anti‐p62 (1:1000, Sigma‐Aldrich, P0067), anti‐Atg7 (1:1000, Cell Signaling Technology, 8558), anti‐p16 (1:1000, Proteintech Group, 10883‐1‐AP), anti‐p21 (1:1000, Proteintech Group, 10355‐1‐AP), anti‐GRP78 (1:1000, Proteintech Group, 11587‐1‐AP), anti‐GRP94 (1:1000, Cell Signaling Technology, 2104), and anti‐glyceraldehyde‐3‐phosphate dehydrogenase (GAPDH) antibody (1:20,000; Sigma‐Aldrich, G9545) were used for immunoblotting. Western blotting was visualized on the Kodak Image Station (Carestream Health Inc., Rochester, NY) and analyzed with the ImageJ software.

### 4.15 Statistical analysis

The data are expressed as means ± standard deviation (SD). Statistical significance (*p* < .05) was evaluated by a one‐way ANOVA combined with Bonferroni's post hoc test. All statistical analyses were performed using a SigmaStat v3.5 software (Systat‐Software, Erkrath, Germany).

## CONFLICT OF INTEREST

None declared.

## AUTHOR CONTRIBUTIONS

A.D.L., E.S.G., J.K.Y., Q.H., Y.Y., and S.P.L. performed research, collected, analyzed, and interpreted data. A.D.L. and H.S.F. conceived the study, designed research, and wrote the manuscript. X.J.J., O.D., U.D., C.T.Z., and D.A.G. conceived the study, designed research, and revised the manuscript. All authors have read and approved the final manuscript.
