# Supplementary figures and images for: Young plasma reverses age‐dependent alterations in hepatic function through the restoration of autophagy
Source: Aging Cell. 2017 Dec 5;17(1):e12708. doi: 10.1111/acel.12708 (PMC5770779; doi:10.1111/acel.12708)

Fig.S1

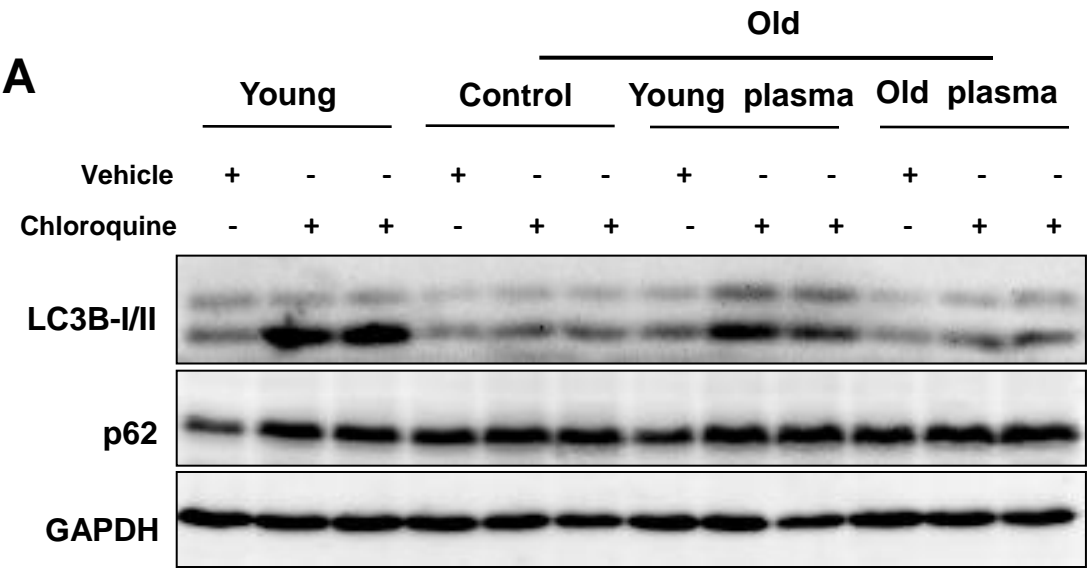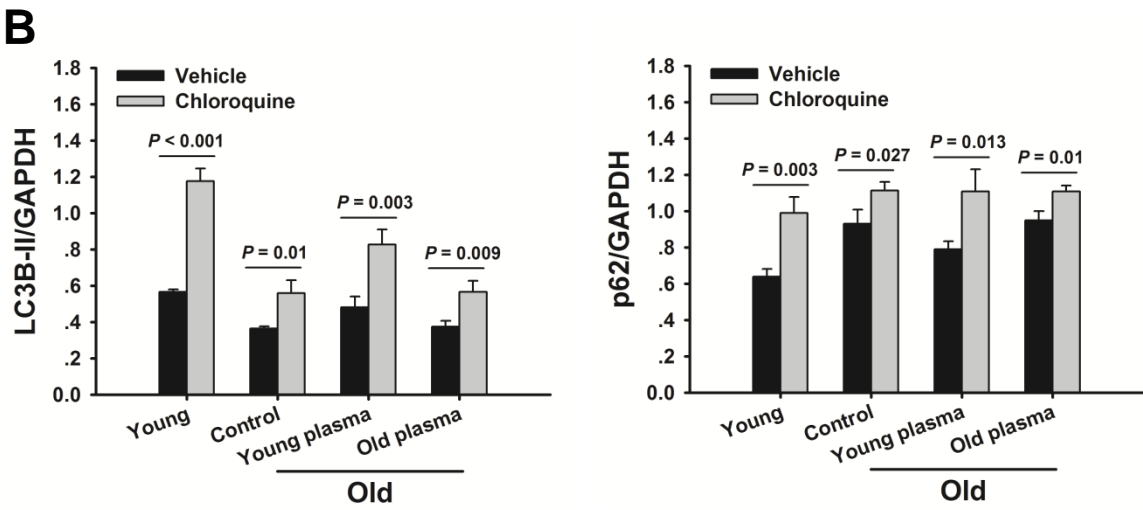

Fig.S2

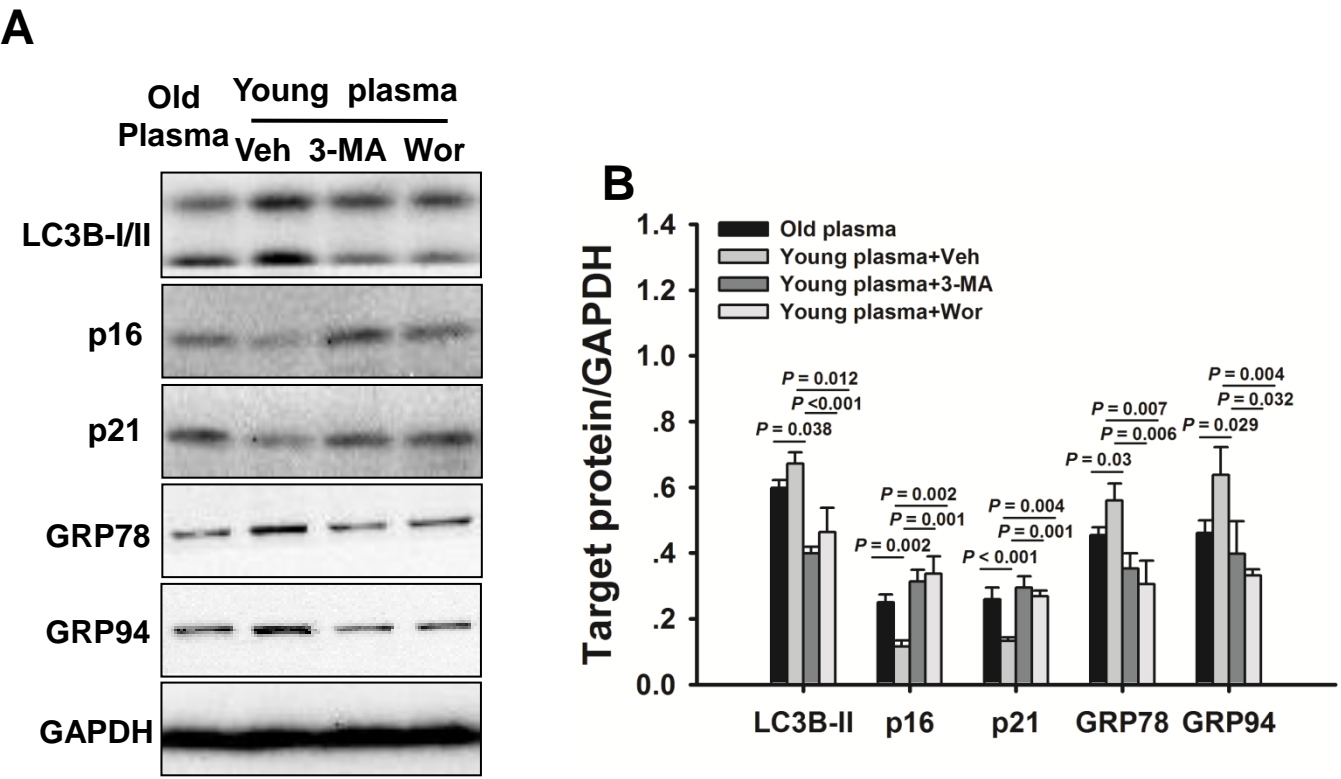

Fig.S3

**A**

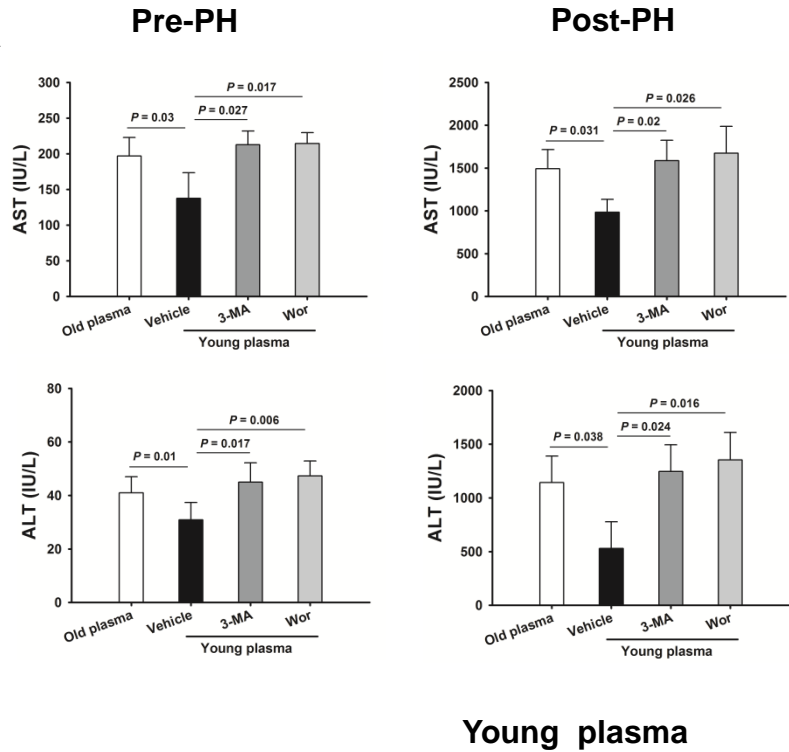

**B**

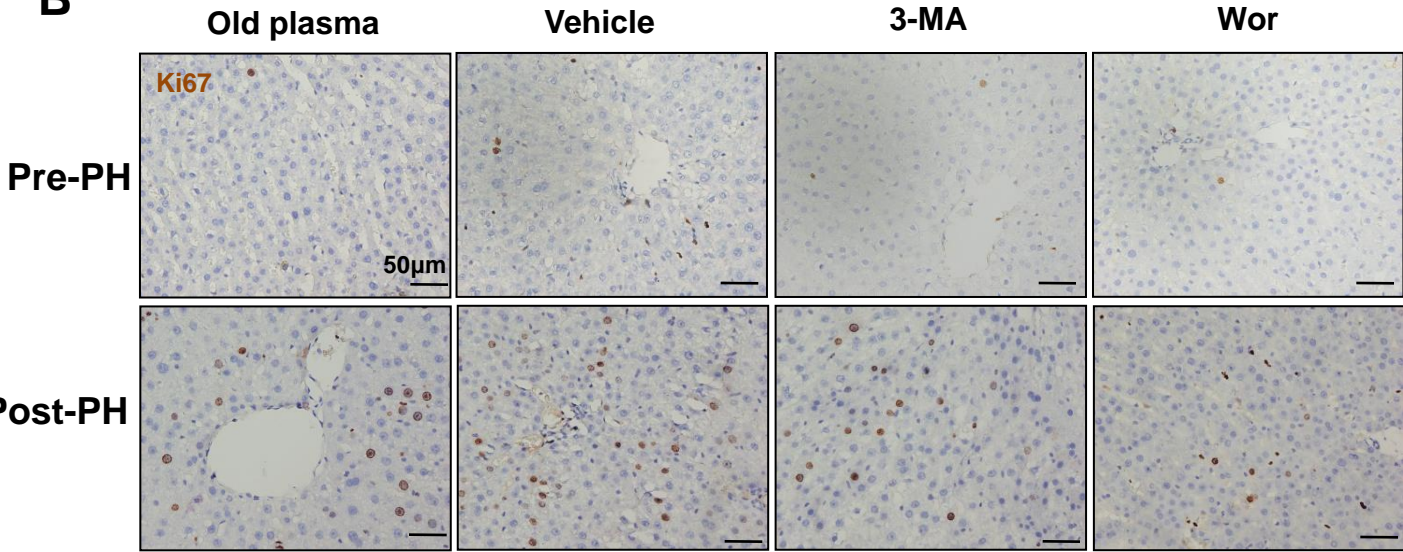

**C**

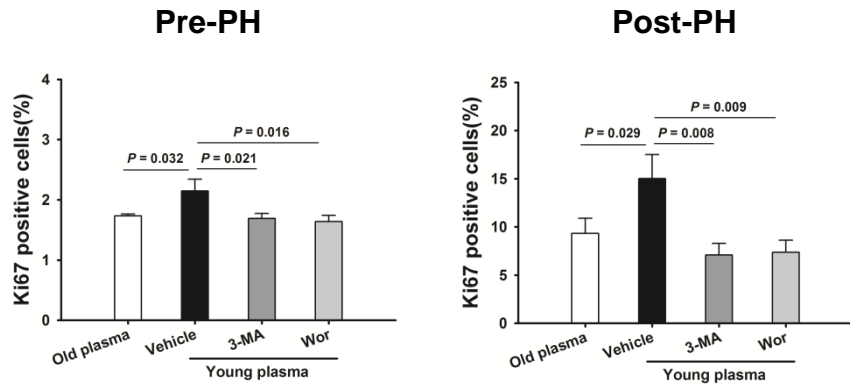

Fig.S4

A

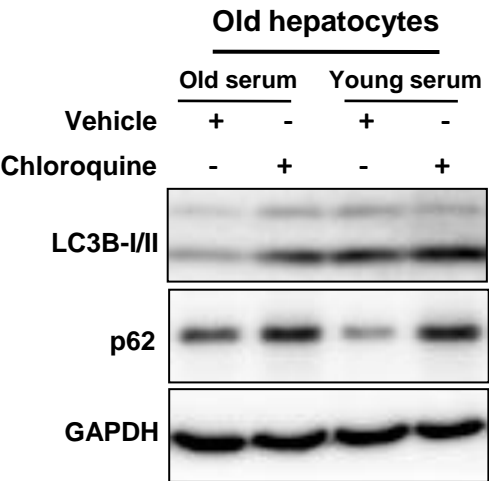

B

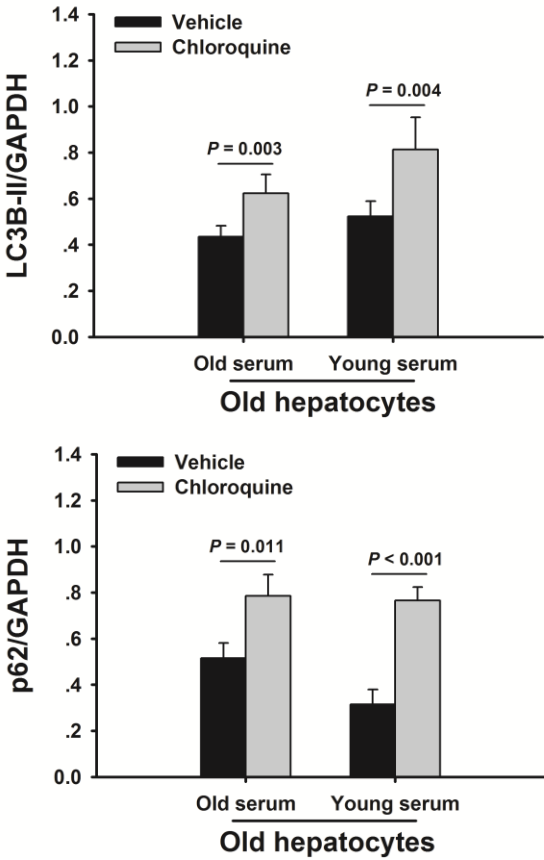

Fig.S5

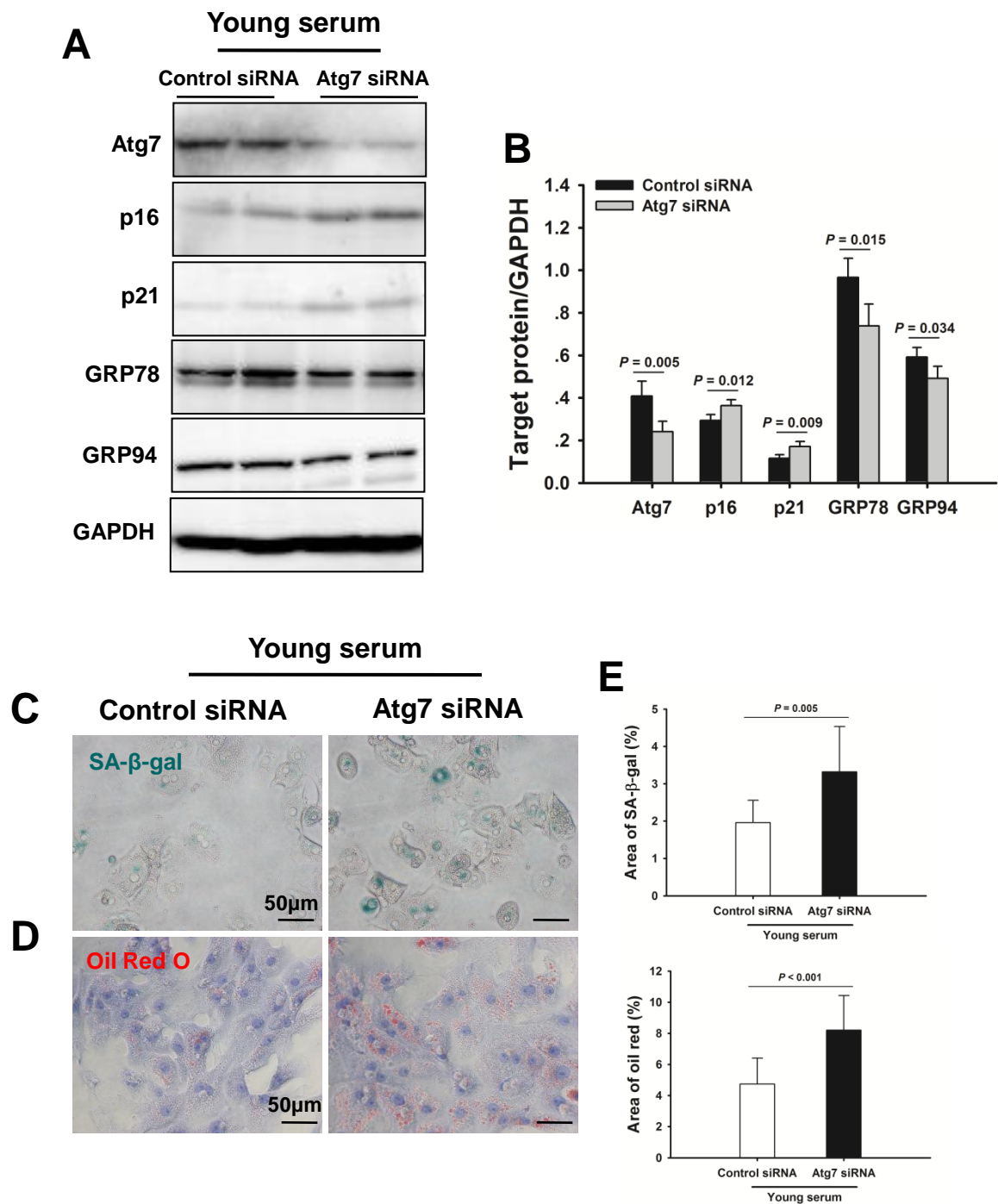

Supplement: Supplementary file 1 [file ACEL-17-na-s001.pdf]
