# Supplementary material for: Young plasma reverses age‐dependent alterations in hepatic function through the restoration of autophagy
Source: Aging Cell. 2017 Dec 5;17(1):e12708. doi: 10.1111/acel.12708 (PMC5770779; doi:10.1111/acel.12708)
Supplement: Supplementary file 2 [file ACEL-17-na-s002.pdf]

Supplemental Figure Legends

Fig. S1 young plasma restores the aging-impaired autophagic flux. Aged rats were treated with pooled young plasma (1 ml, IV) 3 times per week for 4 weeks. (A) To determine autophagic flux, the rats received chloroquine (60 mg/kg, IP) 6 hours before sacrifice. Representative Western blotting of LC3B and p62 protein expression in the presence and absence of chloroquine. (B) Densitometric analysis of LC3B-II and p62. The data are shown as mean ± SD, n = 3 per group.

Fig. S2 Young plasma attenuates aging-induced hepatic senescence and endoplasmic reticulum stress via the restoration of autophagy. Aged rats were treated with pooled young plasma (1 ml, IV) 3 times per week for 4 weeks. Vehicle (Veh), 3-methyladenine (3-MA, 30 mg/kg, IP) or wortmannin (Wor, 0.6 mg/kg, IV) was then given to the rats 3 times per week for 2 weeks before harvest. (A) Representative Western blotting of LC3B, p16, p21, GRP78 and GRP94 protein expression. (B) Densitometric analysis of LC3B-II, p16, p21, GRP78 and GRP94. The data are shown as mean ± SD, n = 3 per group.

Fig. S3 Young plasma rescues aging-induced suppression in liver regeneration via the restoration of autophagy. Aged rats were treated with pooled young plasma (1 ml, IV) 3 times per week for 4 weeks. 3-methyladenine (3-MA, 30 mg/kg, IP) or wortmannin (0.6 mg/kg, IV) was then given to the rats 3 times per week for 2 weeks before harvest. (A) Quantification of serum ALT and AST levels. The data are shown as mean ± SD, n = 3 per group. (B) Representative micrographs exhibiting Ki67-positive hepatocytes in hepatic tissues obtained from pre-partial hepatectomy (PH) and 24

hours post-PH (original magnification, 400 x). (C) The numbers of Ki67-positive hepatocytes were determined. The data are shown as mean ± SD, n = 3 per group.

Fig. S4 Young serum restores autophagic flux in old primary hepatocytes. Old primary hepatocytes were cultured with either young serum or old serum for 72 hours. For autophagic flux, hepatocytes were treated with 10 μM chloroquine for 6 hours before harvest. (A) Representative Western blotting of LC3B and p62 protein expression in the presence and absence of chloroquine. (B) Densitometric analysis of LC3B-II and p62. The experiment was performed in triplicate with similar results. The data are shown as mean ± SD.

Fig. S5 Knockdown of Atg7 prevents the anti-senescence effect of young serum. (A) Representative Western blotting of Atg7, p16, p21, GRP78 and GRP94 protein expression. (B) Densitometric analysis of Atg7, p16, p21, GRP78 and GRP94. The experiment was performed in triplicate with similar results. The data are shown as mean ± SD. (C) Representative micrographs of SA-β-gal staining in hepatocytes with young serum in the absence or presence of Atg7 siRNA (original magnification, 400 x). (D) Representative micrographs of Oil Red O staining in hepatocytes with young serum in the absence or presence of Atg7 siRNA (original magnification, 400 x). (E) Quantitative analysis of SA-β-gal and lipid accumulation. The experiment was performed in triplicate with similar results. The data are shown as mean ± SD.
